# ﻿*Astragalusbashanensis* (Leguminosae), a new species from Central China

**DOI:** 10.3897/phytokeys.219.96916

**Published:** 2023-01-24

**Authors:** Song-Zhi Xu, Qi-Liang Gan, Xin-Wei Li

**Affiliations:** 1 School of Life Science, Nantong University, Nantong, Jiangsu 226019, China Nantong University Nantong China; 2 Zhuxi Qiliang Biological Institute, Zhuxi, Hubei 442300, China Zhuxi Qiliang Biological Institute Zhuxi China; 3 Wuhan Botanical Garden, Chinese Academy of Sciences, Wuhan 430074, Hubei, China Wuhan Botanical Garden, Chinese Academy of Sciences Hubei China

**Keywords:** *Astragalusbashanensis*, Central China, new species, taxonomy

## Abstract

A new species *Astragalusbashanensis*, from western Hubei Province, Central China is described and illustrated. The new species is morphologically similar to *Astragalussinicus* and *A.wulingensis*, but differs from both by its spreading pubescent indumentum on stems and petioles, longer petioles, white bracts, whitish or yellow corolla, longer claw of the keel-petal, hairy pods and smaller seeds.

## ﻿Introduction

*Astragalus* L., consisting of ca. 2500 species, is one of the largest genera of vascular plant (Lewis et al. 2005). *Astragalussinicus* L. (Chinese milk vetch) is native to the Yangtze River Basin of China and has been introduced to many countries as green manure, forage or ornamental plants and became widely naturalised, especially in Japan (Ohashi 2001, 2021; Shimizu 2003). The corolla of *Astragalussinicus* is usually purple, sometimes pink or pure white, but some scholars enlarged the colour range of the corolla to orange (Ho 1993), creamy-yellow or yellow (Xu and Podlech 2010). In the spring of 2022, during field investigations in Zhuxi County, the authors discovered an unknown *Astragalus* species. After comparing with several *Astragalus* species, we found that these specimens with yellow flowers are similar to *A.sinicus* L. and *A.wulingensis* J.X. Li & X.L. Yu by having a prostrate stem, umbellate racemes, outer surface of the calyx sparsely appressed white-pubescent and blackish pod. However, they differ from *A.sinicus* and *A.wulingensis* in habit and morphology of stem, leaf, stipule, bract, flower, fruit and seed (Table 1). Later, we found similar specimens with yellow flowers collected in western Hubei, Central China and stored under the name of *Astragalussinicus* in some herbaria, which were also different from *A.sinicus*. After carefully checking specimens and literature (Ho 1993; Ohashi 2001, 2021; Zhu et al. 2007, 2015; Xu and Podlech 2010), we conclude that the *Astragalus* specimens collected in Zhuxi County and the aforementioned yellow-flowered herbarium specimens stored under the name of *Astragalussinicus* represent a new species placed in Astragalussubgen.Astragalussect.Lotidium Bunge and we describe and illustrate it here.

## ﻿Materials and methods

*Astragalus* specimens were collected in Shennongjia and Zhuxi County of Hubei Province. Comparisons with its relatives were made by consulting specimens stored in PE and HIB, fresh material in the field and some virtual specimen databases (CCAU, KUN, IBK, IBSC, CVH and JSTOR). All morphological characters were measured with dissecting microscopes and were described using the terminology suggested by Harris and Harris (1994).

## ﻿Taxonomic treatment

### ﻿ Astragalus bashanensis

Taxon classificationPlantaeFabalesLeguminosae

Q.L.Gan, X.W.Li & S.Z.Xu sp. nov.

4A5113C9-9857-599B-8610-B041F151FC95

urn:lsid:ipni.org:names:77312598-1

Figs 1 , 2

#### Diagnosis.

*Astragalusbashanensis* Q.L.Gan, X.W.Li & S.Z.Xu is similar to *Astragalussinicus* L. and *A.wulingensis* J.X. Li & X.L. Yu, but the new species can be easily distinguished from both by its spreading pubescent indumentum on stems and petioles, petioles longer than the leaf rachis, white bracts, whitish or yellow corolla, longer claw of the keel-petal, persistent pubescence on both sides of pods and smaller seeds.

**Figure 1.**
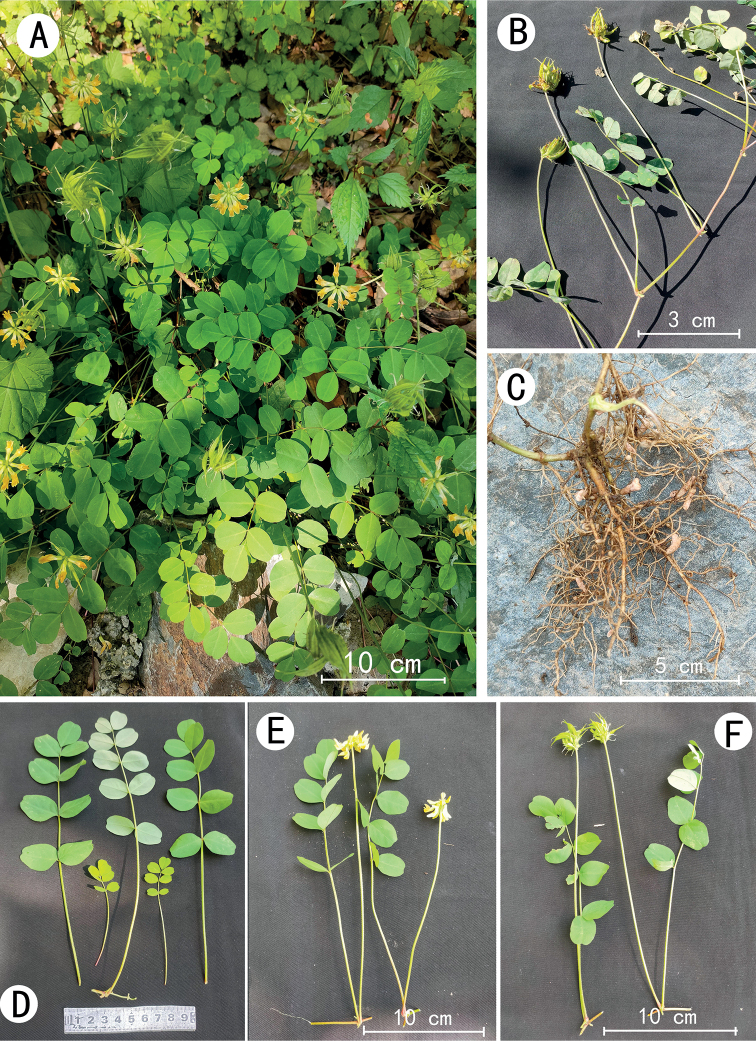
*Astragalusbashanensis* sp. nov. **A** population **B** branches **C** root **D, E, F** leaves.

#### Type.

China. Hubei Province: Zhuxi County, Quanxi Town, Hongyangou Village, on Hengduan Mountain, alt. 840 m, 32°4'8.5"N, 109°39'26.33"E, June 2022, Q. L. Gan 3295 (holotype: PE!; isotype: HIB!).

#### Paratypes.

China. Hubei: Zhuxi County, Jiangjiayan Town, Yanjiajie Village, Piaoshiyan, 3 June 2022, Qi-Liang Gan 3294 (PE!); Quanxi Town, Hongyangou Village, Hengduan Mountain, 7 June 2022, alt. ca. 840 m, Qi-Liang Gan 3295 (PE!); Biaohulinchang, at the foot of Piantou Mountain, alt. 1200 m, Qi-Liang Gan 3287 (PE!); Shennongjia, Dashennongjia, south slopes, alt. 2800 m, flower white, 5 July 1976, Shennongjia Exped. 10718 (PE!, HIB!); Guanmenshan, Shibangou, alt. 2160 m, slopes, 5 August 1976, Shennongjia Exped. 10790 (PE!, HIB!); Guanmenshan, alt. 2150 m, under forests, 5 August 1976, Shennongjia Exped. 10835 (PE!, HIB!); Songluoxiang, Longchahe, Huilongsi, alt. 2000 m, roadside grassland, 2 September 1976, Shennongjia Exped. 22834 (PE!, HIB!).

**Figure 2.**
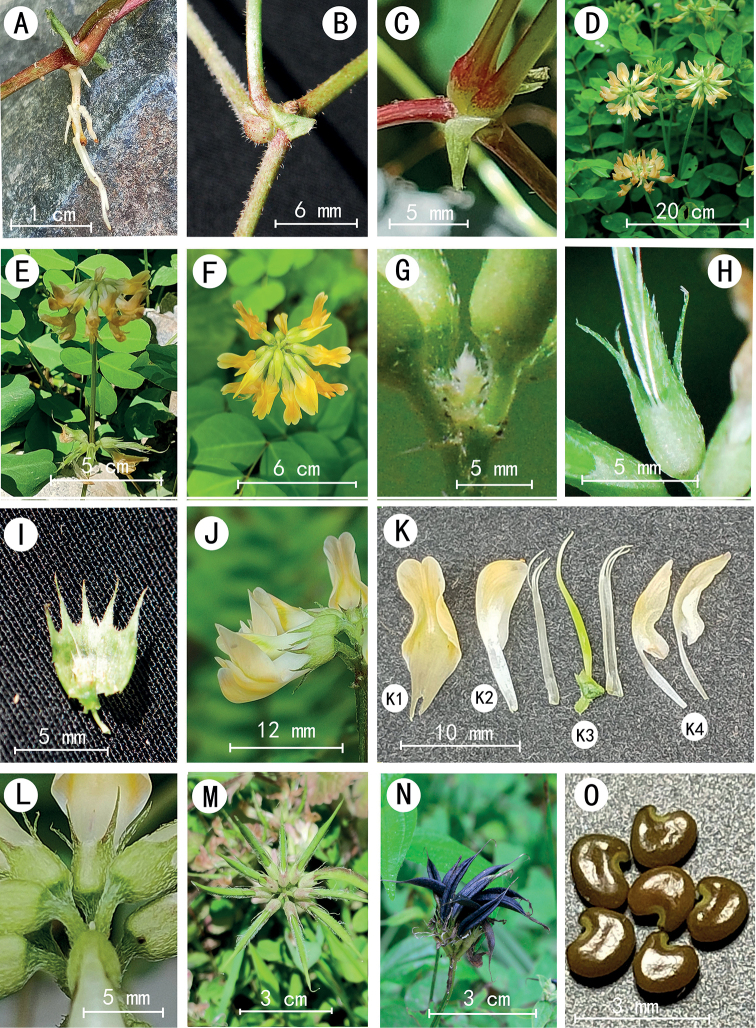
*Astragalusbashanensis* sp. nov. **A** adventitious roots **B** petioles **C** stipules **D** flowering plant **E, F** umbellate racemes **G** bracts **H** calyx **I** opened calyx **J, K** corolla **K1** standard **K2** keel-petal **K3** ovary and stamens **K4** wings **L** pedicels **M** young fruits **N** mature fruits **O** seeds.

#### Description.

Herbs annual or biennial. Primary root slender, 8–12 cm long, yellowish; nodules sparsely on lateral roots. Stem 20–70 cm long, spreading white pubescent; branches from base, prostrate or decumbent, rooting at nodes, internodes 3–8 cm long. Stipules free, triangle-ovate, 3–5 mm long, base 3–4 mm wide, glabrous or with few hairs at apex. Petioles 5–13 cm long, longer than the leaf rachis, spreading-white-pubescent, thickened at base. Leaves odd-pinnate, 7–9-foliolate, rarely even-pinnate 10-foliolate, 5–13.5 cm long, 2.5–6.3 cm wide; rachis sparsely pubescent; leaflets broadly elliptic or broadly obovate, 0.8–3.1 cm long, 0.7–2.4 cm wide, apex emarginate, base rounded or broadly cuneate, margin entire, adaxial surface glabrous, abaxial surface sparsely appressed white-pubescent, the hairs on nerves denser; petiolules less than 1 mm long, densely pubescent. Racemes umbellate, 6–20-flowered, in one, rarely two umbels; peduncles axillary, erect, 10–20 cm long, up to 15–26 cm in fruit, sparsely pubescent; bracts ovate ca. 0.5 mm long, ciliate, white, deciduous after anthesis; pedicels 0.5–1.5 mm long, white-pubescent; flowers spreading or nutant. Calyx tubular, out surface sparsely appressed white-pubescent; tube ca. 3 mm long; lobes 5, subulate, 2.6–3.2 mm long, lower two shorter than the tube, upper three longer than or as long as the tube. Corolla whitish, creamy-yellow, yellowish or deep yellow; standard obovate, 10–13 mm long, 4–5 mm wide, apex emarginate, base broadly cuneate with short claw; wings 8–10 mm long, claw scarcely shorter than the limb, limb oblique-oblong; keel-petal 7–9 mm long, limb crescent-shaped, base auriculate, claw ca. 2/3 of limb length. Stamens (9) + 1, white, ca. as long as the keel-petal. Ovary narrowly linear, both sides white pubescent; style filiform, glabrous. Pod linear, lateral oblate and green when young; mature pod swelling into boat-like, blackish, 2.5–3 cm long, ca. 4 mm wide, both sides persistent white-pubescent, apex with a thicker beak ca. 1 cm long. Seed dark green-brown, lustrous, orbicular-reniform or reniform, 1.5–1.8 mm long.

#### Phenology.

Flowering from late May to early July; fruiting from mid-June to late August.

#### Distribution and habitat.

Populations of *Astragalusbashanensis* are known from Quanxi Town, Zhongfeng Town, Longba Town, Jiangjiayan Town, Piantoushan National Forest Park and Baguashan Provincial Nature Protection Area of Zhuxi County and it is also widely distributed in Shennongjia National Forest Park, western Hubei, Central China. It grows in roadside grassland, on mountain slopes, forest edges or under forest canopy at elevations from 600 to 2160 m.

#### Etymology.

The specific epithet is derived from the type locality of the new species. Bashan is an abbreviation for Dabashan or Daba Mountains.

#### Vernacular name.

Ba Shan Huang Qi (Chinese).

#### Conservation assessment.

During our field investigations in 2021 and 2022, many populations of *A.bashanensis* have been found in Zhuxi County and Shennongjia Forest Region. The numbers of individuals of each population ranges from dozens to thousands. In addition, it is distributed along roadsides as a weed. We believe that it should have a much wider distribution than what is now known. Due to its wide distribution range and large population size, *A.bashanensis* is here recommended as Least Concern (LC) (IUCN 2022).

#### Results.

*Astragalusbashanensis* is most similar to *A.sinicus* and *A.wulingensis* in having prostrate stems, simple hairs, umbellate racemes, outer surface of calyx sparsely appressed white-pubescent and blackish and boat-like pods, but it can be easily distinguished from both by its spreading-pubescent indumentum on stems and petioles (vs. ascending-pubescent in *A.sinicus*; appressed-puberulent in *A.wulingensis*), long petioles (5–13 cm long), longer than the leaf rachis (vs. much shorter than the leaf rachis), white bracts (vs. green or flushed purplish), corolla whitish or yellow (vs. purple, pink to white or yellowish flushed purplish at apex), longer claw of the keel-petal (vs. much shorter), both sides of pods persistent pubescent (vs. glabrous or glabrate) and smaller seeds 1.5–1.8 mm long (vs. 2–3 mm long). The diagnostic features between *A.bashanensis*, *A.wulingensis* and *A.sinicus* are summarised in Table 1.

**Table 1.** Morphological comparisons of *Astragalusbashanensis*, *A.wulingensis*, and *A.sinicus*.

Characters	*A.wulingensis*	*A.bashanensis*	*A.sinicus*
Stems	30–80 cm long	20–70 cm long	10–30 cm long
Stipules	ciliate	glabrous or with few hairs at apex	ciliate
Petioles	3–5 cm long, much shorter than the leaf rachis	5–13 cm long, longer than the leaf rachis	0.5–4 cm long, much shorter than the leaf rachis
Indumentum on stems and petioles	sparsely appressed-pubescent	spreading-pubescent	sparsely ascending-pubescent
Leaflets	1.5–2.8 × 0.8–1.1 cm	0.8–3.1 × 0.7–2.4 cm	0.5–1.7 × 0.3–1.3 cm
Bracts	green or flushed purplish	white	green or flushed purplish
Calyx	tubular, ca. 7 mm long	tubular, 5–6 mm long	campanulate, 3–5 mm long
Lobes of calyx	subulate, 3–4 mm long	subulate, 2.6–3.2 mm long	lanceolate, 1–2 (–3) mm long
Corolla colour	white or yellow, apex flushed purplish	whitish, creamy yellow, yellowish or deep yellow	purple, pink to pure white
Claw of keel-petal	ca. as long as the limb	ca. 2/3 length of the limb	ca. 1/3 length of the limb
Wings	10–13 mm long, claw as long as the limb	8–10 mm long, claw scarcely shorter than the limb	7–11 mm long, claw ca. 1/3 length of the limb
Pods	3–4 cm long, glabrous	2.5–3 cm long, both sides persistent-pubescent	1.2–2 cm long, glabrous or glabrate
Seeds	orbicular-deltoid, 2–2.2 mm long, red-brown	orbicular-reniform or reniform, 1.5–1.8 mm long, dark green-brown	reniform, 2–3 mm long, green-brown

## Supplementary Material

XML Treatment for Astragalus bashanensis
